# Collectanea Medica, Consisting of Anecdotes, Facts, Extracts, Illustrations, Queries, Suggestions, &c.

**Published:** 1818-11

**Authors:** 


### COLLECTANEA MEDICA,

CONSISTING OF

### ANECDOTES, FACTS, EXTRACTS, ILLUSTRATIONS, QUERIES, SUGGESTIONS, &c.

RELATING TO THE

*History or the Art of Medicine, and the Auxiliary Sciences.*

Quicquid agunt medici, nostri farrago libelli.

### *Change of Colour of the Skin of an American Indian.*

THE following account of this singular phenomenon is by Dr. Emery Bissel, of Clinton, New York, and is published in the Transactions of the Medico-Physical Society. We have thought it more appropriate for insertion in this department of our Journal, than in that which we devote to the consideration of subjects more intimately connected with the practice of medicine.

The subject of it is a man named Samuel Adams, of the Brotherton tribe of Indians, living in the vicinity of New York. He is now ninety years of age, and has been gradually becoming white for the last thirty years. Considering his age, he is remarkably vigorous and healthy; having been able during the warm season of the year to attend to the culture of his land. He is in perfect possession of his mental faculties.

The skin began to undergo a change of colour at the age of sixty, very soon after an attack of acute rheumatism. The first appearance of it was a small white patch near the pit of the stomach. Soon after this, spots of the same colour occurred on different parts of the body and limbs, gradually increasing both in size and number.

He was at this time greatly alarmed, and visited the different mineral springs in the surrounding country, in the hope of removing a colour so odious to him by ablution in their waters. Finding these measures ineffectual, and being convinced that no danger was to be apprehended from a white skin, he relinquished the idea of regaining his native colour, and (to use his own words) "patiently submitted to become like the white men in every thing, but their dishonesty."

Since it commenced, the change has been constantly going on, though not in a very uniform manner; at times progressing very rapidly, at others remaining nearly stationary. These irregularities seem never to have been influenced by the season of the year. The original colour, at this time, remains only on the forehead, forepart of the face and neck, with a few small patches on the arms.

The skin on the body and lower extremities is perfectly white, soft, and pliable, having nothing of a dull chalky appearance; neither does it possess the livid hue generally observable in the skins of Albinos, but is remarkably fine and clear. It has, in fact, a much nearer resemblance to the delicate skin of a female than to that of a man of ninety.

In those parts which have become white, perspiration has always been somewhat less than in the others. They are also more sensible; being much more susceptible of the effects of heat and cold, and often blistered by the heat of the sun. They are likewise very tender, and bleed much when cut or lacerated, and heal with difficulty. Neither the pigmentum nigrum, nor hair, has undergone any other change than is incident to old age. The latter is moderately grey, and the eye presents no other than the dim, cloudy appearance generally found in the eyes of old people. His vision is now tolerably perfect, and he asserts that it was never in the least impaired until the age of eighty. Except perspiration and cutaneous sensibility, Dr. Bissel could not discover that a single function of the system had ever suffered any derangement in consequence of this state of the skin.

The secretions and excretions are natural, and he now labours under no other disease than a trembling of the limbs, occasioned by a paralytic attack about thirty years since; with a slight cough, that attends him in the winter.

He has had no children since the age of sixty; consequently the case furnishes us with no facts how far a change of colour in the skin of the parent might affect the complexion of the offspring.

He affirms, that he never was before in his life afflicted with any cutaneous disease, except the itch, and that but twice; that his complexion was much darker than is common among those of his own race,—or, as he says, "he was a very black Indian."

Now we are engaged on this curious subject, we shall transcribe the short account of a similar fact related by Dr. Rush, in the *American Philosophical Transactions*, vol. iv. p. 295.

"In a certain Henry Moss, who lately travelled to this city (Philadelphia), the change from a black to a natural white flesh-colour began about five years ago, at the ends of his fingers, and has extended gradually over the greatest part of his body. The wool, which formerly perforated the cuticle, has been changed into hair. No change in the diet, drink, dress, employment, or situation, of this man had taken place previously to this change in the skin."

We refer such of our readers as may feel particularly interested with this subject, to a Tract published at Leipzig, in 1785, by M. Bose, entitled "*de Mutato per morbum Colore Corporis Humani.*"

---

*On Ingurgitation.* (From the Journal Complementaire du Dict. des Sciences Medicales. 1e. Cahier.)

By ingurgitation is generally understood, the state of a cavity which is filled so as to overflow. But this term, very little used, although particularly expressive, is almost solely applied to the stomach surcharged with food and drink.

Ingurgitation, when habitual, tends to distend the stomach, sometimes even to ten times its natural size: we have one preserved, which will contain eighteen pints of fluid. The person to whom it belonged was a dragoon, a great eater, who was shot for desertion. He was previously supplied with food and drink, so that he might take it until he became satiated. On opening his body, still warm, his stomach was found to fill nearly the whole abdominal cavity.

Persons who live on aliments which supply but little matter for the nourishment of the body, are obliged to eat in large quantities; they daily induce ingurgitation, which terminates in unnatural dilatation of the stomach.

Ingurgitation is more dangerous from an ill quality of the substance which constitutes it, than from its quantity. Thus a *Lazaroni*, lying on his back, enjoys himself with sipping iced water, (*aqua nevata*,) as long as it will enter his stomach, (the quantity taken is usually five or six bottles.) When he is well swelled out, he falls asleep, wakes to urine, and sleeps again: after three or four hours, he gets up, and walks about. We once tried how much macaroni one of these *Lazaroni* would eat. It was at a fair, before a great concourse of people, drawn together to witness the spectacle: he emptied a large kettle filled with it. The quantity cost seventeen shillings.

Nothing is more common than ingurgitation with grapes during the vintage; and it is but rarely that any ill arises from this practice, although they are often eaten until not another can be swallowed. This, however, is not the case when the ingurgitation is with wine, and more particularly ardent spirits. A foolish species of vanity formerly induced men to contest with their fellow guests at table the ability for excessive drinking. The Romans enumerated the number of glasses which should be drank, and often imposed a double or triple quantity on those who did not observe the rules of the feast. The Emperor Claudius was in the habit of swallowing wine and luxurious viands until he was obliged to be carried from table; he was then laid on his back, and vomiting induced by tickling his throat with a feather. Vitellius repeated this disgusting scene three or four times a day, to have the pleasure of again gorging himself with oysters, of which he was excessively fond. Many accidents are related to have ensued from those kind of *orgies*, against which Cicero thundered in vain, both in the senate and at the tribunal.

Our ancestors, the Gauls and Celts, were insatiable drinkers; and Poultier, in his history, has not neglected to speak of the consequences resulting from their almost continual ingurgitation of wine and beer.

Will it be credited that, at a certain period, it was the fashion in France, even with courtiers, to resort to a tavern, and not to leave it but with the stomach filled with wine, and the nose crammed with snuff? Certain weak and delicate noblemen have paid dearly for endeavouring to equal in intemperance robust and habitual courtiers: many a family has deplored the loss of an heir from this cause.

Count Bassompierre contributed more than any of his contemporaries to the reputation which the French had of being able to drink with any nation in Europe, when, after a sumptuous dinner, well moistened with wine, he took leave of the Swiss, to whom he had been sent as an envoy. As he mounted his horse they presented to him some wine in a goblet containing several quarts. "Ah, ah! gentlemen," said he, "I will drink it; but here, I have my own glass:" so pulling off his boot, he filled it to the brim, and drank it to their health. The noble envoy performed his journey on horseback, the stomach, without doubt, emptying itself gradually by vomiting as he rode along. This is what generally happens under such circumstances to strong persons, and who, they say, are habitually inclined to vomit. The shaking of the exercise produces a kind of gentle vomiting, which establishes the equilibrium of the stomach, and prevents the ill consequences of drunkenness. It is sufficient, in many instances of ingurgitation, to stoop forward, to produce a flow of liquids and half-digested food. We sometimes see, as Peyer and several others have observed, a sort of rumination take place, which, rendering the overcharge gradually, relieves the stomach in a gentle manner from the inconvenience which would otherwise result from the practice.

M. de Bassompierre, on the occasion we have mentioned, drank Rhenish wine of a hundred years. This is worthy of notice. If the ingurgitation of his lordship had been with the heavy red wine of the Rhone, or the fuming liquor of the south of France, matters would not have passed off so easily. It is in the latter cases we witness that brutal drunkenness, which the ancients called *plumbeam et indecoram ebrietatem;* a kind of inebriety which light, acidulous, and cold wines, with difficulty produce.

We have, in the North, seen men, gorged with beer, laid over a cask on their belly, to make the liquor escape from the stomach; which supposes a relaxation of the cardia that really exists, since the œsophagus itself is sometimes full, and justifies the proverb—*he is filled to the throat.* A child is sometimes made to tread on the abdomen of an individual in this state, to force out the beer from the stomach; without which, it was said, his life was endangered. There have been many instances of rupture of the stomach and bladder from the above cause.

Ingurgitation is a vice of all ages, but it has not been considered as disgraceful in all countries. In ancient Judea it was a common practice to produce immediate vomiting, that the pleasure of drinking might be immediately repeated; which habit is common, at the present time, in some parts of Germany. We shall terminate this article with the relation of an instance we witnessed in the latter country.

It is a custom, at Treves, to make a yearly pilgrimage to a high mountain situated near the left bank of the Moselle. Several families often join in a party, and carry with them provisions, of which they eat on their arrival at an agreeable spot on the top of the mountain. Troops of musicians place themselves at the head of these parties, playing on their instruments: but a very small recompense in money is paid them, but the provisions, and particularly the wine, in an abundant quantity, are left for them. One of these fellows told us, that he had several times made three journeys in a day, and, being completely drunk on the first, we thought it impossible that he could still eat and drink; but he said he would be prepared for it as soon as he wished, which was effected by producing vomiting. When we arrived at the place, he found a new society, before which he began to play, after having performed his disgusting operation.—Percy and Laurent.

---

*On the Medicinal Properties of Lauro-cerasus.* (From vol. 27 of the *Dictionnaire des Sciences Medicales.*)

The medicinal properties of *lauro-cerasus* are far from being as well ascertained as its poisonous qualities. Some physicians have described it as a tonic and stomachic; but it is by others, with more propriety, considered a sedative: it has been praised as one of the most powerful alteratives, and even as antisyphilitic. It appears to increase the secretion of urine. The leaves, reduced to powder, excite sneezing, even in those accustomed to the use of snuff.

Cameron asserts that he has cured the most obstinate hepatic obstructions by an infusion of laurel-leaves. Cataplasms of millet-farine, with the same infusion, or the distilled water of the plant, appeared to diminish, and in some cases even to cure, scirrhous and carcinomatous tumors. Vogel states, that the external and internal use of the distilled water was productive of temporary benefit in those cases.

Baylis considered it as the most powerful remedy for removing too great tenacity of the blood, and of considerable service in hectic fever.

The following observation of M. Cevasco (*Bibl. Medic.* 1808, vol. xix. p. 231,) seems to prove that distilled laurel-water may be sometimes useful to diminish too great irritability of the heart, and favour an increase, at the same time, of the action of the absorbent vessels.—A soldier, twenty years of age, suffered for a long time from violent throbbing of the heart, which prevented his using violent exercise. On being obliged to join his regiment, his complaints became much increased, and he was soon afterwards transmitted to the military hospital at Genoa. The throbbing of the heart was so great that it might be distinguished through his dress. Hæmoptysis, and tumefaction of the lower extremities, shortly made their appearance; for which he was twice bled, without experiencing any relief. Twenty drops of distilled laurelwater* were exhibited daily in three pints of barley-water, and the quantity was gradually increased to fifty drops. The pulse soon became less frequent and resistent; the hæmoptysis ceased, and the throbbing nearly subsided; and his general strength so much improved in the space of a month, that he was dismissed fit for military duty.

M. Cevasco adds, that he has witnessed the antiphlogistic powers of this remedy in many cases of peripneumony, enteritis, and angina; and that he is convinced it possesses powerful and useful medicinal qualities.

Linnæus (*Amoen. Acad.* vol. iv. p. 40, et *Mat. Med.*) says, that it was frequently used in Holland in cases of pulmonary consumption. The Essays published by M. Majendie on the use of *prussic acid* in that disease, make us presume that the infusion, or distilled water, of laurel might be employed with advantage with the same intention, as it contains a considerable portion of prussic acid.

---

### *On the Use of Colchicum in Gout.*

The following observations on the above remedy are transcribed from the Journal of the Royal Institution. We need only mention that they are by Sir Everard Home, and are also deduced from his personal experience, to convince our readers of their importance.

"In the communications which I made to the Royal Society, upon the use of the Vinum Colchici in Gout, in the year 1817, I was desirous of putting an end to the quackery respecting that medicine, and to restore it to the place it held in the pharmacopœia in the time of the Greek physicians; at the same time that I explained an improvement in its preparation, from which I have myself received so much benefit, that I was desirous of giving my fellow sufferers an opportunity of partaking of it.

"The Philosophical Transactions being a work set aside for investigations and recording new facts, not for practical essays, I contented myself with stating what was new; leaving to the practitioners in medicine to put my observations to the test, and diffuse the benefits of this mode of employing the medicine.

"I am now induced, by the numerous applications that are made to me for further information on the subject, to send the following observations to you, with a request that you will give them a place in the Journal of the Royal Institution; by which means they will be more generally diffused than through the medium of the Philosophical Transactions.

The facts on which I wish to lay particular stress are the following:—

1st. That the vinum colchici, when received into the circulation in small doses, produces no deleterious effects, and only brings on

* It is not mentioned how prepared.

the same symptoms as when taken into the stomach, which go off in similar periods of time.

2nd. That the deposit which is separated from the infusion by keeping, when given by itself in the dose of a few grains, produces inflammation and ulceration on the coats of the stomach and intestines.

" 3d. That the infusion removes the paroxysm of gout equally readily whether given without the deposit as with it.

" 4th. The infusion, when clear of the deposit, can be taken in doses of sixty or seventy minims, without producing any disturbance in the stomach, increasing any of the secretions, or bringing on an irregularity of the pulse,—effects which commonly occur when that dose is given with the deposit mixed with it.

" 5th. That sixty minims is the smallest dose that can be depended upon for the complete removal of a paroxysm of gout.

" These are the facts which I communicated to the Royal Society, and which are confirmed by an experience of seventeen months upon myself and many other individuals. In some constitutions a dose of seventy minims may be required; and that dose I know can be taken with safety, as I have taken it myself, and found no sensible effect from it, further than a slight nausea, and that the attack of gout was carried off.

" The advantage of taking the colchicum in this form is, that, being milder, the dose necessary for the cure can always be employed without injury to the stomach, even in states of the greatest debility.

" The vinum colchici, without the deposit, has another advantage; for, as it does not irritate the stomach, it does not require being compounded with other medicines to defend the stomach from its effects.

" The buds of the colchicum should be collected after the disappearance of the leaves, which happens in July, and before the appearance of the flower, early in September."

---

We quote the following passage from the admirable work of Pinel on *Mental Alienation*, as relating to the account we inserted in the last number of this Journal on the management of lunatics in the hospital at Avignon.

" I have examined, with the most scrupulous attention, the effects produced on lunatics by the use of iron chains, and the results of its abolition, and I no longer doubt of the superior advantages of a *mild* mode of restraint. The same lunatics, who, kept in chains for a long series of years, had continued in a state of fury, walked about tranquilly under the use of gentle means of restraint, and talked with composure to those they met; while, before that, they could not be approached without extreme danger."

